# Exteroceptive and Interoceptive Body-Self Awareness in Fibromyalgia Patients

**DOI:** 10.3389/fnhum.2017.00117

**Published:** 2017-03-13

**Authors:** Camila Valenzuela-Moguillansky, Alejandro Reyes-Reyes, María I. Gaete

**Affiliations:** ^1^Centro de Estudios de Argumentación y Razonamiento, Facultad de Psicología, Universidad Diego PortalesSantiago, Chile; ^2^Instituto de Sistemas Complejos de ValparaísoValparaíso, Chile; ^3^Escuela de Psicología, Universidad Santo TomásConcepción, Chile; ^4^Department of Psychiatry and Mental Health, Universidad de ChileSantiago, Chile

**Keywords:** exteroception, interoception, body awareness, chronic pain, fibromyalgia

## Abstract

Fibromyalgia is a widespread chronic pain disease characterized by generalized musculoskeletal pain and fatigue. It substantially affects patients' relationship with their bodies and quality of life, but few studies have investigated the relationship between pain and body awareness in fibromyalgia. We examined exteroceptive and interoceptive aspects of body awareness in 30 women with fibromyalgia and 29 control participants. Exteroceptive body awareness was assessed by a body-scaled action-anticipation task in which participants estimated whether they could pass through apertures of different widths. Interoceptive sensitivity (IS) was assessed by a heartbeat detection task where participants counted their heartbeats during different time intervals. Interoceptive awareness was assessed by the Multidimensional Assessment of Interoceptive Awareness (MAIA). The “passability ratio” (the aperture size for a 50% positive response rate, divided by shoulder width), assessed by the body-scaled action-anticipation task, was higher for fibromyalgia participants, indicating disrupted exteroceptive awareness. Overestimating body size correlated positively with pain and its impact on functionality, but not with pain intensity. There was no difference in IS between groups. Fibromyalgia patients exhibited a higher tendency to note bodily sensations and decreased body confidence. In addition, the passability ratio and IS score correlated negatively across the whole sample, suggesting an inverse relationship between exteroceptive and interoceptive body awareness. There was a lower tendency to actively listen to the body for insight, with higher passability ratios across the whole sample. Based on our results and building on the fear-avoidance model, we outline a proposal that highlights possible interactions between exteroceptive and interoceptive body awareness and pain. Movement based contemplative practices that target sensory-motor integration and foster non-judgmental reconnection with bodily sensations are suggested to improve body confidence, functionality, and quality of life.

## Introduction

“It is as if all of my joints were locked and I am caught inside, as though imprisoned in a kind of body or an envelope that has padlocks inside, like doors that I cannot open.”

Fibromyalgia patient interview

(Valenzuela-Moguillansky, 2012).

The present study was performed to assess two aspects of body awareness in fibromyalgia patients: exteroception and interoception. Fibromyalgia is a chronic disease characterized by generalized musculoskeletal pain and fatigue. It is diagnosed based on the presence of at least 11 of 18 tender point sites on digital palpation (Wolfe et al., 2010; see Wolfe and Häuser, 2011 for an up to-date discussion). Dysfunction in processing and modulation of noxious stimuli by the central nervous system, and hyperactivity of the sympathetic nervous system are purportedly involved (Cohen et al., 2000; Martinez-Lavin, 2007; McEwen and Kalia, 2010; Bellato et al., 2012), as are psychiatric conditions including anxiety, panic disorder, post-traumatic stress disorder, and general depression (Epstein et al., 1999; Bair et al., 2003; Raphael et al., 2004; Van Houdenhove et al., 2005; Arnold et al., 2006; van Houdenhove and Luyten, 2006). The etiology and pathogenesis of fibromyalgia remain unclear. Pregabalin, duloxetine, and milnacipran are approved pharmacological therapies, but their use is limited by side effects, and not all patients experience improvement (Ablin and Buskila, 2010). The prevalence of fibromyalgia is between 1.6 and 2.1% in Europe and the United States (Wolfe et al., 1995, 2013; Perrot et al., 2011), and 1–2% in Chile, where the present study was performed (Trujillo-Lira, 2007).

Fibromyalgia impacts an individual's relationship with their bodies. As illustrated by the opening quote, the body becomes an obstacle. While pain loci might change from patient to patient or from 1 day to another, pain and fatigue are felt over the whole body (Valenzuela-Moguillansky, 2013; Calsius et al., 2015). The body becomes a salient, unfamiliar presence that prevents daily activities and affects quality of life, social relationships, and functionality (Burckhardt et al., 1993; Arnold et al., 2008). Valenzuela-Moguillansky (2013) investigated the bodily experience in fibromyalgia over the course of a pain crisis from a phenomenological perspective. As pain increased, a series of changes in patients' body perception were observed. They described changes in the perception of their body size and its relationship with space: they felt their body becoming larger and as though space was shrinking. These results are in line with those of Akkaya et al. (2012) who report that body image is disrupted in fibromyalgia. Moreover, patients with fibromyalgia exhibit a higher frequency of falls and loss of balance despite an absence of inflammatory joint damage (Jones et al., 2009; Meireles et al., 2014), suggesting that a sensorimotor aspect of body awareness is also affected.

Body awareness involves aspects differentially conceptualized by discipline or researcher (Gallagher, 2000; de Preester and Knockaert, 2005; de Vignemont, 2010). In the present work, we refer to the notions of exteroceptive awareness, interoceptive sensitivity, and interoceptive awareness. Exteroceptive body awareness (or “the body schema”) refers to the implicit knowledge we have of our body in relation to space and movement. It results from the integration of multimodal exteroceptive signals (e.g., vision, sound, touch), vestibular and proprioceptive systems, and voluntary motor systems. Even though the term “body schema” is more commonly used, we refer to exteroceptive body awareness to highlight the relationship with interoceptive body awareness (Harshaw, 2015). Both terms emphasize the internal representation we have of our body and posture in guiding action and are thus interchangeable. Previous work has revealed alterations of exteroceptive body awareness in other chronic pain syndromes (e.g., Schwoebel et al., 2001, 2002; Bray and Moseley, 2011). Whether exteroceptive body awareness is affected in fibromyalgia has not been evaluated.

Interoception refers to the perception of our internal state. Originally introduced by Sherrington (1906), this term was linked to visceral sensitivity, meaning the ability to detect signals coming from our “internal milieu.” This term was redefined by Craig (2002) as the sense of the physiological condition of the body beyond the viscera, thus expanding the notion and positioning it in the afferent pathway of the autonomic nervous system. Under this view, afferent signals from the various body tissues that contribute to the regulation of physiological parameters constitute “a basis for the subjective evaluation of one's condition,” allowing better understanding of organic body function and its relationship to mental and emotional experiences. In recent years, diverse lines of research including emotional and social cognition, mental health, and sense of the bodily self have incorporated interoception in investigations as a key element of the mind-body relationship. It is therefore of interest to investigate whether interoception is altered in persons with fibromyalgia.

The notion of interoception has multiple aspects. Harshaw (2015) presented a detailed taxonomy of interoceptive dysfunction; however, the defined terms are liable to ambiguous and interchangeable use. Further, they are based on assessment methods rather than a clear conceptual construct. For example, using the heartbeat detection task, a widely used method to measure interoception, enhanced interoception associated with emotional liability, anxiety, lower pain thresholds, and lower pain tolerability (Schandry, 1981; Ehlers and Breuer, 1992; Cameron, 2002; Eley et al., 2004; Pollatos et al., 2012), while diminished interoception associated with depression and alexithymia (Pollatos et al., 2009; Herbert et al., 2011; Terhaar et al., 2012). Under a different framework, enhanced interoception is related to non-judgmental acceptance of bodily sensations and a sense of self grounded in experiencing physical sensations in the present moment (Mehling et al., 2009). Mehling et al. (2012) elaborated a self-report questionnaire called the Multidimensional Assessment of Interoceptive Awareness (MAIA) to assess this type of interoception. With the heartbeat detection task, we refer to *interoceptive sensitivity*, while with the MAIA we refer to *interoceptive awareness*.

Given the impact fibromyalgia has on patients' bodily experience and functionality, we consider it relevant to investigate whether exteroceptive and interoceptive aspects of body awareness are altered in fibromyalgia patients in greater detail. Developing from previous work, we hypothesize that fibromyalgia patients exhibit disrupted exteroceptive body awareness and disrupted interoceptive sensitivity (IS). As fibromyalgia has been related to traits such as anxiety and depression, each associated with heightened and diminished IS, respectively, we will not propose a specific *a priori* hypothesis, but rather explore responses in this area. In addition, we propose that interoceptive awareness is decreased in fibromyalgia patients, hypothesizing a reduced sense of self grounded in experiencing physical sensations, and reduced ability to regulate emotional responses based on a connection with one's own body, in situation of chronic pain.

Finally, as exteroceptive and interoceptive body awareness are constructs that point to different aspects of an integrated experience of the bodily self, we consider relevant to assess whether these are related. Tsakiris et al. (2011) found that people with low IS are more prone to body illusions that involve ownership of a foreign body part, concluding that interoceptive awareness modulates the online integration of multisensory body stimuli. Moseley et al. (2008) found that inducing the illusion of ownership of a rubber hand decreases the temperature of participants' “disowned” hand, suggesting that changes in body schema impact homeostatic regulation of physiological parameters (see Harshaw, 2015 for a comprehensive review and additional examples). We hypothesize that there is a relationship between exteroceptive and interoceptive body awareness in both fibromyalgia and control participants.

Investigating in greater detail which aspects of body awareness are altered in fibromyalgia patients, and determining how this occurs might improve therapeutic strategies and their evaluation, as well as encourage reflection on the relationship between pain and body awareness.

## Methods

### Design and participants

This comparative, cross-sectional study was performed in a laboratory setting. Fifty-nine female participants aged 22–71 years were included. Thirty fibromyalgia patients were recruited from the Valparaíso (Chile) Regional Fibromyalgia Association, and 29 healthy controls were recruited among patients' immediate social environment, aiming for similarity between groups in socioeconomic, cultural, and educational aspects. Fibromyalgia was diagnosed according to the American College of Rheumatology (ACR) criteria. Patients were included if they were over 18 years of age, reported pain equal to or >4(on a scale from 0 to 10), experienced pain at least 4 days per week and over at least the previous 6 months, consented to participate, and demonstrated the ability to read and understand the informed consent form and questionnaires. In addition, patients who received medical treatment for pain were asked to have a constant medication dosage during the 2 weeks prior to inclusion. Exclusion criteria included treatment for major depression; history of neurological conditions such as epilepsy, stroke, organic brain impairment, and dementia; autoimmune diseases or diseases affecting the autonomic nervous system; cardiovascular disease; diabetes mellitus; pain <6 months; attentional or intellectual deficits; eating disorders; use of drugs or excessive alcohol use; pregnancy; and amputees or a physical disability. Additionally, controls were excluded if they had any chronic pain condition. Due to low prevalence, male fibromyalgia patients were not recruited. There were no significant differences in age, body mass, or educational level between the two groups (Table 1).

**Table 1:** **Summary of the demographic characteristics of fibromyalgia patients and the participants of the control group**.

	**Fibromyalgia group (*****n*** = **30)**				**Control group (*****n*** = **29)**						
***Variables***	***Min***.	***Max***.	***M***	***SD***	***Min***.	***Max***.	***M***	***SD***	***t-z***	***p-value***	***d***
Age (years)	22	71	46.77	12.66	22	61	43.52	10.97	1.052	0.30^a^	0.27
Weight (Kg)	53	110	66.15	11.58	50	85	66.62	10.47	−0.164	0.74^b^	−0.04
Size (m)	1.50	1.70	1.60	0.05	1.50	1.86	1.63	0.07	−1.761	0.08^a^	−0.46
BMI (kg/m^2^)	19.47	39.44	26.00	4.46	19.72	35.56	25.29	4.38	0.614	0.41^b^	0.16
Duration of the pain (months)	21	540	173.33	164.60	0	0	−	−	−	−	−
Current pain intensity (0 to 10)	0	9	5.00	2.26	0	5	0.28	1.07	−6.277	0.00^b^	2.67
Educational level	*n*	%			*n*	%					
Primary	0	0			2	6.9			−1.46	0.143^c^	
Secondary complete	1	3.3			1	3.4			−0.02	0.983^c^	
Secondary incomplete	1	3.3			2	6.9			−0.63	0.529^c^	
Technical-professional	13	43.3			11	37.9			0.42	0.673^c^	
College degree	10	33.3			7	24.1			0.78	0.435^c^	
Postgraduate studies	5	16.7			6	20.7			−0.39	0.693^c^	

*n, Sample size; M, Mean; SD, Standard Deviation; BMI, body mass index; a, t-test; b, Mann-Whitney U-Test; c, Z-Test; d, Cohen's d*.

The Institutional Bioethics Committee of the University of Valparaíso (Chile) approved the study. Each participant received an information sheet and provided written, informed consent to participate.

## Materials

### Clinical assessments

#### The fibromyalgia impact questionnaire (FIQ)

The Fibromyalgia Impact Questionnaire (FIQ) is a 19-item self-report questionnaire that covers three domains: “physical function,” “overall impact,” and “symptoms.” The physical function domain contains 10 items that use a 4-point Likert scale with a response set ranging from “always” to “never.” The overall impact domain contains two items measured by number of days in the previous week. The symptoms domain contains 7 items using 100-mm anchored visual analog scales. The FIQ has been used in large-scale clinical trials for fibromyalgia therapies (Williams and Arnold, 2011). We used an adaptation of a validated Spanish translation of the FIQ (Esteve-Vives et al., 2007) to assess fibromyalgia symptoms. Internal consistency of the FIQ, measured by Cronbach's alpha coefficient, was estimated at 0.93.

#### The symptoms impact questionnaire (SIQ)

The Symptoms Impact Questionnaire (SIQ) is identical to the FIQ but does not refer to fibromyalgia and is used to compare fibromyalgia patients to other groups (Friend and Bennett, 2011). We used the SIQ to identify symptoms of discomfort in the control group.

#### The short form of the brief pain inventory (BPI)

The short form of the Brief Pain Inventory (BPI) is a two-dimensional, self-report questionnaire that assesses pain intensity (*Severity* dimension) and the impact of pain on functioning (*Interference* dimension). Answers are given across a 10-point Likert scale (0 meaning no severity or interference and 10 meaning worse intensity or complete interference). The BPI is recommended for use in clinical settings to monitor the severity and impact of general pain (Williams and Arnold, 2011). We used a validated Spanish translation of the BPI (Cleeland, 1991) in fibromyalgia and control groups. Internal consistency of the BPI was estimated at a Cronbach's alpha coefficient of 0.97 for the overall score, with 0.95 and 0.97 for the *Severity* and *Interference* dimensions, respectively.

#### The depression anxiety stress scale (DASS-21)

The Depression Anxiety Stress Scale (DASS-21) is a three-dimensional, 21-item, self-report questionnaire that assesses depression, anxiety, and stress. Answers are given according to a 4-point Likert scale (0 meaning “this statement does not describe what happened to me during the last week” and 3 meaning “this statement describes much of what happened to me during the last week”). We used a Spanish translation validated in a Chilean population (Antúnez and Vinet, 2012) to assess depression, anxiety, and stress in fibromyalgia and control groups. Internal consistency was estimated at a Cronbach's alpha of 0.96 for the total score, with coefficients of 0.93, 0.84, and 0.91 for the dimensions of *Depression, Anxiety*, and *Stress*, respectively.

Participants were also asked to report their current pain intensity on a scale from 0 to 10.

### Exteroceptive body awareness

#### Body-scaled action task

The body scale action task was performed following the protocol of Guardia et al. (2010). Fifty-one apertures varying from 35 to 78 cm were projected onto a wall in random fashion (constant stimuli method, E-prime software). The video projector was positioned sufficiently far (4.3 m) to allow the projection zone to reach the floor and present a 2-m-high aperture such that the projection was similar to a real door. The participant stood upright behind the video projector, 4.8 m from the wall on which the aperture was projected. Participants were instructed to imagine themselves walking at a normal speed and to say, without performing the action, whether they would be able to pass through the presented aperture without turning sideways, pressing a button for “yes” or “no.” Each aperture was presented four times for a total of 204 randomly ordered trials. When the task was completed, the experimenter measured the participant's shoulder width. As performed by Guardia et al., we calculated participants' perceptual threshold as the aperture for which they gave a 50% positive response rate (“Yes, I can walk through without turning sideways”).

We calculated the slope of the psychometric curve as follows:

$$\begin{array}{l} \text{Answer }=1/1+\exp^{\left(-\text{k}\left(\text{c}-\text{aperture}\right)\right)} \end{array}$$

Where *c* is the aperture corresponding to the perceptual threshold and *k* is the slope of the curve around the point *c*. The slope indicates the discriminability of the participants: steep and shallow slopes correspond to good and poor discrimination, respectively. Dividing the perceptual threshold by the participants' shoulder width, we calculated the perceived passability ratio (π_p_). The passability ratio is an index that indicates the estimate that a person makes of her body size in relation to the width of her shoulders. Thus, if the index is equal to 1, the perceptual threshold is equal to the width of shoulders of the person. The larger the index, the greater the width that the person needs to estimate that she passes through the aperture. Warren and Whang (1987) used a similar task to show that the passability ratio in healthy subjects is 1.16.

### Interoceptive sensitivity

#### Heartbeat detection-task

The heartbeat-detection task was performed following the protocol of Tsakiris et al. (2011). Participants were asked to silently count their heartbeats during an interval determined by two auditory cues while their heartbeats were monitored using a three-electrode electrocardiogram (ECG, Biopac MP36R). There were four different intervals of 75, 45, 35, and 25 s, presented in random order per participant, who was then asked to report the number of heartbeats counted at the end of each interval.

IS was estimated as the mean heartbeat perception score:

$$\begin{array}{l} \text{IS score}=1/4\text{S}(1-[|\text{recorded heartbeats}—\text{counted}\\ \text{ heartbeats}|]/\text{recorded heartbeats}) \end{array}$$

Accordingly, the IS score ranges from 0 to 1, with higher scores indicating smaller differences between counted and recorded heartbeats.

### Interoceptive awareness

#### The MAIA

The MAIA is a 32-item self-report questionnaire composed of eight subscales, evaluating the following per category. *Noticing*, awareness of uncomfortable, comfortable, and neutral body sensations; *Not distracting*, not ignoring or distracting oneself from sensations of pain or discomfort; *Not worrying*, not worrying or experiencing emotional distress with sensations of pain or discomfort; *Attention regulation*, ability to sustain and control attention to body sensation; *Emotional awareness*, awareness of the connection between body sensations and emotional states; *Self-regulation*, ability to regulate psychological distress by attention to body sensations; *Body listening*, actively listening to the body for insight; and *Trusting*, experiencing one's body as safe and trustworthy.

Items are answered on a Likert scale, with six levels of ordinal responses coded from 0 (never) to 5 (always). We translated the MAIA questionnaire to Spanish and evaluated the Spanish version's psychometric properties (Valenzuela-Moguillansky and Reyes-Reyes, 2015). It was used in the present study to assess interoceptive body awareness in fibromyalgia and control groups. In terms of reliability, a Cronbach's alpha value of 0.90 was estimated for the total score, while subscales ranged from 0.21 to 0.85: Noticing (α = 0.74), Not distracting (α = 0.21), Not worrying (α = 0.39), Attention regulation (α = 0.85), Emotional awareness (α = 0.84), Self-regulation (α = 0.85), Body listening (α = 0.85), and Trusting (α = 0.78).

### Procedure

Prior to the experimental session, participants were contacted by telephone to agree to an appointment and register personal information (age, educational level, duration of the pain, intensity of the pain, description of other symptoms, medications, and other illnesses). On arrival, participants were provided with written information about the experiment, and informed consent was obtained. Next, they answered four questionnaires: the FIQ/SIQ^1^, BPI, DASS-21, and MAIA. They were then seated in a comfortable chair, ECG electrodes were placed, and the heartbeat detection task commenced. Two training trials were performed prior to four experimental trials (described above). At the end of the heartbeat perception task, a short interview was given about the participants' performance. Between the heartbeat detection and body-scaled action tasks, we registered participants' cardiac activity during 5 min of rest and 5 min of a cognitive stress task (objective of a parallel study). ECG electrodes were removed, participants asked to stand up-right and the body-scaled action task was performed (described above). The experimental session lasted approximately 75 min.

### Statistical analysis

Student's *t*-tests for independent samples were used to compare the means of variables between fibromyalgia and control groups. Correlations between variables were assessed with the Pearson coefficient. Mann-Whitney and Spearman tests were applied for non-normal distributions and non-homogeneous between group variances. The Shapiro-Wilk test was used to test normality. The *Z*-test was used to compare proportions of two independent samples. A two-tailed hypothesis test was performed using a significance level of 0.05.

The Expectation Maximization (EM) method was used to impute missing data with a likelihood function based on a Student t distribution. Little's Missing Completely at Random (MCAR) test was applied over the data set.

Analyses were performed using IBM SPSS Statistics 22 (IBM Corp, 2011) and with StataSE (StataCorp, 2015).

## Results

### Group comparisons

There were no significant differences between the fibromyalgia and control groups in age, weight, height and body mass index (Table 1). Education level was similar in both groups (all *p* > 0.05).

### Clinical assessments

The FIQ scores of the fibromyalgia group were higher than the SIQ scores of the control group. The S*everity* and *Interference* scores of the BPI were higher in fibromyalgia patients, as were the *Depression, Anxiety*, and *Stress* dimensions of the DASS-21 (Table 2). Distribution of pain and the frequency at each location are shown in Table 3.

**Table 2:** **Comparison of clinical assessment of the participants with fibromyalgia and control group**.

***Variables***	**Fibromyalgia group (*****n*** = **30)**				**Control group (*****n*** = **29)**						
	***Min***.	***Max***.	***M***	***SD***	***Min***.	***Max***.	***M***	***SD***	***t-z***	***p-value***	***d***
FIQ/SIQ	20.50	90.96	59.72	19.51	.00	54.72	18.45	13.40	9.497	0.000^a^	2.47
BPI severity	7	40	20.23	6.20	0	25	4.17	6.50	−5.973	0.0001^b^	2.53
BPI interference	7	65	37.57	16.00	0	40	4.62	9.58	−6.161	0.0001^b^	2.45
DASS-21 total	3	56	26.00	14.78	0	19	6.69	5.01	5.26	<0.001^b^	1.75
DASS-21 depression	0	21	7.63	6.53	0	8	1.90	2.24	3.87	<0.001^b^	1.17
DASS-21 anxiety	0	17	7.53	4.58	0	5	1.55	1.50	5.55	<0.001^b^	1.75
DASS-21 stress	0	20	10.83	5.17	0	10	3.24	3.02	5.13	<0.001^b^	1.79

*FIQ, Fibromyalgia impact questionnaire; SIQ, Symptoms impact questionnaire; BPI, Brief pain inventory; DASS-21, Depression, anxiety, stress scale. n, Sample size; M, Mean; SD, Standard Deviation; a, t-test; b, Mann-Whitney U-Test; d, Cohen's d*.

**Table 3:** **Distribution and frequency of pain in fibromyalgia patients**.

**Anterior**				**Posterior**			
**Body zone**	**Side**	**Frequency**		**Body zone**	**Side**	**Frequency**	
Head		4	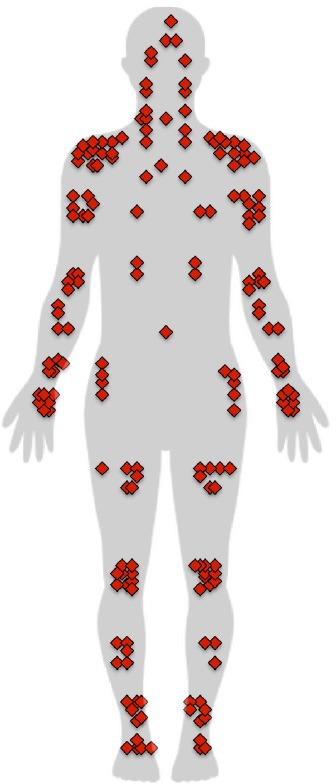	Head		5	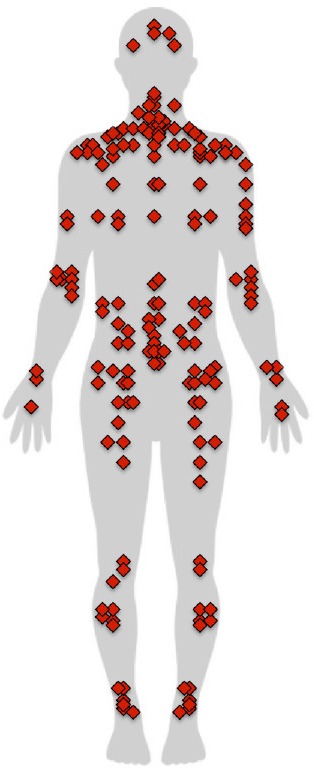
Face		1		Cervical	Middle	13	
Jaw	Right	2			Right	5	
	Left	2			Left	6	
Neck	Middle	1		Shoulders	Right	7	
	Right	5			Left	5	
	Left	4		Upper arm	Right	5	
Shoulders	Right	13			Left	2	
	Left	10		Elbow	Right	6	
Upper arm	Right	7			Left	7	
	Left	8		Wrist	Right	3	
Elbow	Right	5			Left	2	
	Left	5		Hand	Right	2	
Forearm	Right	4			Left	1	
	Left	4		Upper thorax	Middle	3	
Wrist	Right	5			Right	6	
	Left	4			Left	6	
Hand	Right	7		Lower thorax	Middle	4	
	Left	7			Right	4	
Chest	Middle	1			Left	4	
	Right	3		Lumbar region	Middle	14	
	Left	4			Right	7	
Ribs	Right	2			Left	7	
	Left	2		Sacrum region	Middle	1	
Belly	Middle	1			Right	2	
Hip	Right	4			Left	2	
	Left	5		Buttocks	Right	8	
Thigh	Right	6			Left	8	
	Left	7		Thigh	Right	4	
Knee	Right			9	Left	3	
	Left	10		Knee	Right	2	
Shin	Right	5			Left	3	
	Left	3		Calf	Right	5	
Ankle	Right	6			Left	5	
	Left	6		Ankle	Right	6	
Foot	Right	5			Left	6	
	Left	3					

### Exteroceptive body awareness

**Figure 1:**
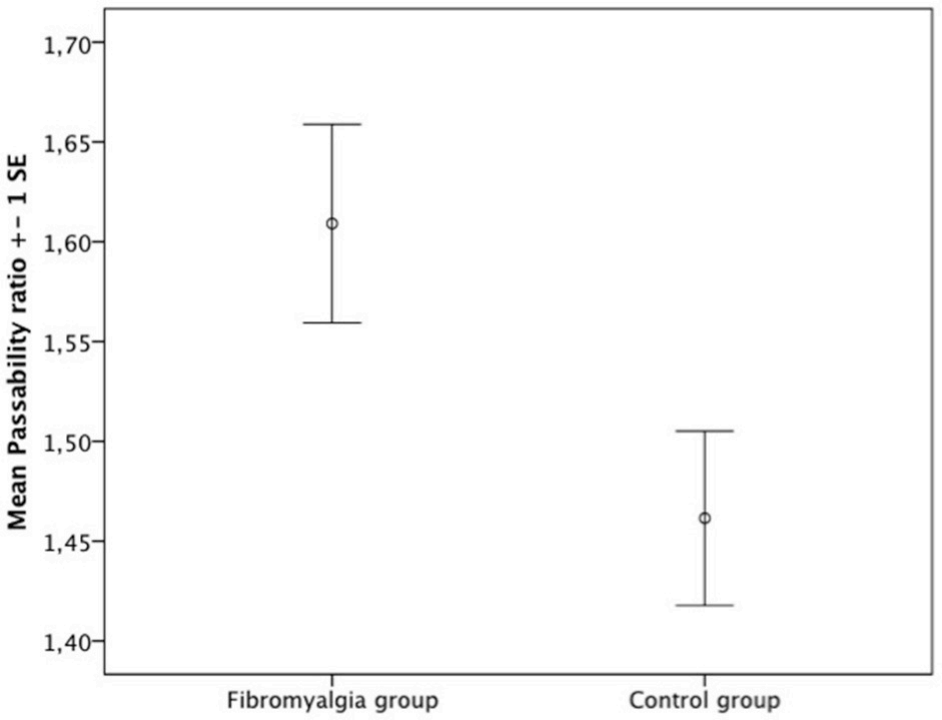
**Comparison between groups of mean passability ratio**.

The passability ratio was higher in the fibromyalgia group (Figure 1, mean π_FM_ ± SD: 1.61 ± 0.26; mean π_C_ ± SD: 1.46 ± 0.23; *t* = 2.209, *p* = 0.03; *d* = 0.61). We compared the means of the psychometric curves slopes of both groups and found no differences in discriminability (Table 4, mean slope _FM_ ± SD: −0.77 ± 0.41; mean slope _C_ ± SD: −0.91 ± 0.43; *U* = 346.5; *z* = −1.342, *p* = 0.18; *d* = 0.33).

**Table 4:** **Summary of the slope, critical aperture, shoulder width and the passability ratio (π_***p***_) in the two groups**.

	**Fibromyalgia group**				**Control group**						
	***Min***.	***Max***.	***M***	***SD***	***Min***.	***Max***.	***M***	***SD***	***t-z***	***p-value***	***d***
Slope	−1.93	−0.29	−0.77	0.41	−1.89	−0.23	−0.91	0.43	−1.342	0.18^b^	0.33
Critical aperture (cm)	43.6	73.0	56.09	8.16	34.50	73.0	53.77	8.50	1.067	0.29^a^	0.28
Shoulder width (cm)	32	40	35.59	2.35	32	40	36.46	2.35	−1.394	0.169^a^	−0.37
Passability ratio (π_*p*_)	1.23	2.15	1.61	0.26	0.91	1.87	1.46	.23	2.231	0.030^a^	0.61

*M, Mean; DS, Standard Deviation; a, t-test; b, Mann-Whitney U-Test; d, Cohen's d*.

A correlation analysis was performed to test the relationship between the passability ratio and clinical variables (FIQ/SIQ, BPI, current pain score, and DASS-21). Correlations were observed between the passability ratio and FIQ/SIQ score (*r* = 0.364, *p* = 0.006) and the *Interference* dimension of the BPI (*r* = 0.334, *p* = 0.012). No correlation was found between the passability ratio and BPI severity or the current pain score. There was no correlation between the passability ratio and the DASS-21. We also tested the relationship between the passability ratio and pain duration but did not find a significant correlation. Though not significant, a progressive increment of the mean ratio was observed when pain duration was stratified in three categories: 0 months (absence of pain), 1–96 months, and 97–540 months (mean π_0 months_ = 1.48 ± 0.25, mean π_21 to 96 months_ = 1.55 ± 0.247, mean π_97 to 540 months_ = 1.60 ± 0.305).

### Interoceptive sensitivity

No difference between groups was observed for the IS score (mean _FM_ ± SD: 0.49 ± 0.31, mean _C_ ± SD: 0.50 ± 0.26; *t* = −0.169; *p* = 0.867; *d* = −0.035).

To assess relationships between IS and clinical variables, we performed a correlation analysis over the whole sample between the IS score and the FIQ/SIQ, BPI, and DASS-21 results. There was a negative correlation between IS and the *Depression* dimension of the DASS-21 (Table 5). Examining each group, a negative correlation was found between the IS score and the *Depression* dimension, *Stress* dimension, and total DASS-21 score among participants with fibromyalgia. In the control group, a positive correlation was observed between the IS score and the *Anxiety* dimension of the DASS-21.

**Table 5:** **Pearson correlation coefficient between the interoceptive sensitivity (IS) score and the DASS-21 in the fibromyalgia and control groups**.

**Variables**	**IS score**		
	**Whole Sample**	**Fibromyalgia group**	**Control group**
FIQ/SIQ	−0.129	−0.294	0.023
BPI *Severity*	−0.094	−0.245	0.013
BPI *Interference*	−0.112	−0.304	0.131
DASS-21 Depression	−0.298^*^	−0.431^*^	−0.108
DASS-21 Anxiety	−0.051	−0.201	0.450^*^
DASS-21 Stress	−0.184	−0.403^*^	0.117
DASS-21 Total score	−0.203	−0.394^*^	0.157

* *p < 0.05*.

### Interoceptive awareness

The MAIA questionnaire scores are displayed in Table 6. Participants with fibromyalgia registered lower scores in the dimensions *Not distracting* (*F* = 5.153, *p* = 0.027, $\eta_{p}^{2}$ = 0.084) and *Trusting* (*F* = 12.113, *p* = 0.001, $\eta_{p}^{2}$ = 0.178) and higher scores in *Noticing* (*F* = 6.031, *p* = 0.017, $\eta_{p}^{2}$ = 0.097).

**Table 6:** **Descriptive statistics of the MAIA dimensions according to the fibromyalgia and control groups**.

**Dimensions**	**Fibromyalgia group**					**Control group**							
	***Min***.	***Max***.	***M***	***Md***	***SD***	***Min***.	***Max***.	***M***	***Md***	***SD***	***F***	***p***	**$\eta_{p}^{2}$**
Noticing	6	20	15.57	16.00	3.70	3	20	12.82	13.00	4.78	6.031	0.017	0.097
Not distracting	0	11	5.97	6.50	3.02	2	13	7.71	8.00	2.83	5.153	0.027	0.084
Not worrying	0	15	7.17	7.00	3.46	1	15	8.64	9.50	3.27	2.785	0.101	0.047
Attention regulation	5	33	18.27	18.50	7.53	1	32	19.36	20.50	7.91	0.289	0.593	0.005
Emotional awareness	0	25	18.43	19.50	6.53	4	25	19.11	21.00	5.63	0.176	0.676	0.003
Self-regulation	0	20	9.40	10.00	5.45	3	20	11.89	12.00	4.92	3.329	0.073	0.056
Body listening	0	14	6.67	6.00	4.19	0	13	6.43	6.50	3.99	0.049	0.826	0.001
Trusting	1	15	7.87	8.00	3.32	0	15	10.96	12.00	3.46	12.113	0.001	0.178
MAIA total	35	127	89.33	90.50	24.22	43	147	96.93	97.00	25.03	1.379	0.245	0.024

*M, Mean; DS, Standard Deviation; $\eta_{p}^{\text{2}}$, Partial eta squared; Md, Median*.

A negative correlation was found between the total MAIA score and the FIQ/SIQ (Table 7). The dimension *Noticing* correlated positively, while *Not distracting, Not worrying, Self-regulation*, and *Trusting* correlated negatively.

**Table 7:** **Pearson correlation coefficient between the MAIA and the FIQ, BPI, and DASS-21 considering the whole sample**.

		**BPI**		**DASS-21**			
**MAIA**	**FIQ/SIQ**	***Severity***	***Interference***	***Depression***	***Anxiety***	***Stress***	**Total**
Noticing	0.362^**^	0.168	0.170	0.202	0.385^**^	0.336^**^	0.327^*^
Not distracting	−0.329^*^	−0.296^*^	−0.359^**^	−0.392^**^	−0.210	−0.334^*^	−0.345^**^
Not worrying	−0.392^**^	−0.251	−0.327^*^	−0.449^**^	−0.363^**^	−0.414^**^	−0.445^**^
Attention regulation	−0.076	−0.207	−0.178	−0.196	−0.013	−0.072	−0.107
Emotional awareness	−0.109	−0.092	−0.113	−0.221	−0.079	−0.087	−0.143
Self-regulation	−0.299^*^	−0.287^*^	−0.363^**^	−0.292^*^	−0.205	−0.258^*^	−0.276^*^
Body listening	−0.088	−0.046	−0.136	−0.189	−0.043	−0.003	−0.087
Trusting	−0.442^**^	−0.416^**^	−0.439^**^	−0.247	−0.379^**^	−0.338^**^	−0.343^**^
MAIA Total	−0.289^*^	−0.297^*^	−0.364^**^	−0.370^**^	−0.194	−0.242	−0.296^*^

* *p < 0.05*,

** *p < 0.01*.

The *Severity* dimension of the BPI correlated negatively with the total MAIA score. The *Not distracting, Self-regulation*, and *Trusting* dimensions of the MAIA correlated negatively with BPI severity. The total MAIA score exhibited a negative correlation with *Interference*, as did the individual dimensions of *Not distracting, Not worrying, Self-regulation*, and *Trusting*.

There was a negative correlation between total DASS-21 score and that of the MAIA. The total MAIA score correlated negatively with the *Depression* dimension of the DASS-21. *Noticing* on the MAIA correlated positively with *Anxiety, Stress*, and the total DASS-21 score. *Not distracting* correlated negatively with *Depression, Stress*, and total DASS-21 score. *Not worrying* correlated negatively with *Depression, Anxiety*, and *Stress* on the DASS-21, as well as the total score. *Self-regulation* correlated negatively with *Depression* and the total DASS-21 score. *Trusting* correlated negatively with *Anxiety, Stress*, and total DASS-21 score.

### Exteroceptive and interoceptive body awareness

To evaluate whether there is a relationship between exteroceptive and interoceptive body awareness, we tested correlations between a) the passability ratio and IS score and b) the passability ratio and MAIA. The passability ratio and the IS score correlated inversely (*r* = −0.291, *p* = 0.05), as did the passability ratio and *Body listening* dimension of the MAIA (*r* = −0.355, *p* = 0.001), although it did not correlate with any other MAIA dimension or its total score.

## Discussion

The aims of the present study were to evaluate exteroceptive and interoceptive self-body awareness of persons suffering from fibromyalgia and to assess whether there is a relationship between exteroceptive and interoceptive body awareness. Our hypotheses were (a) fibromyalgia patients have disrupted exteroceptive body awareness, overestimating their body size; (b) fibromyalgia patients present disrupted IS; (c) fibromyalgia patients have diminished interoceptive awareness compared with control subjects; and (d) there is a relationship between exteroceptive and interoceptive self-body awareness within the whole sample.

### Exteroceptive body awareness

Consistent with our first hypothesis, the passability ratio of the body-scaled action-anticipation task was higher among fibromyalgia patients. They overestimated the passability of an aperture relative to their shoulder width, suggesting a disruption in their exteroceptive body awareness. To our knowledge, this is the first study to report such a result. The lack of difference in the slope of the psychometric curve compared to controls suggests that the difference in the passability ratio is not due to group differences in the ability to perform the task. This result extends on our previous findings (Valenzuela-Moguillansky, 2013), where fibromyalgia patients described feeling a larger body over the course of a pain crisis.

Notably, the passability ratio in our control group (1.46) was larger than ratios obtained in previous studies employing the body-scaled action task. Warren and Whang (1987) obtained a passability ratio of 1.16 in controls, whiles 2010 and 2012 studies by Guardia et al. reported 1.15 and 1.14, respectively. Group differences between the samples might explain this disparity. Warren and Wang included male undergraduates, while Guardia et al. (2010, 2012) included young women with a mean age around 24. The mean age of the women participating in the present study was 45. Increase in hip width with age is generally larger than that of shoulder width. Normalization of the critical opening (the aperture for which participants gave a 50% positive response rate) is performed by shoulder rather than hip width, so it is plausible that the passability ratio increases with age in women. There was no difference in age between the two groups; thus, this difference in the passability ratio compared to previous studies has no bearing on the results with respect to our hypotheses.

Our results show that body size overestimation correlates positively with the *Interference* dimension of the BPI but not the *Severity* dimension or current pain intensity. This suggests that the change in exteroceptive body awareness is not due to moment-to-moment incorporation of sensory (nociceptive) changes, as proposed by Schwoebel et al. (2001, 2002), where we would have expected the passability ratio and pain intensity to correlate due to an impact of pain on functionality. Pain-related fear and fear-avoidance behavior have been extensively reported in different chronic pain conditions (e.g., Jensen and Karoly, 1992; Asmundson et al., 1997; Crombez et al., 1999; Leeuw et al., 2007; Wideman et al., 2009). Pain-related avoidance behavior affects range of movement and muscular strength, changing the motor response patterns (Vlaeyen and Linton, 2000). Disrupted body awareness in fibromyalgia patients might be the result of such a process. This is in line with findings by Moseley (2004) and Peltz et al. (2011). Moseley applied the hand laterality task in complex regional pain syndrome (CRPS) patients to test: (a) if chronic disuse is responsible for a delay in hand recognition, reaction times should be proportional to duration of symptoms and (b) if a guarding response contributes to the delay in hand recognition, reaction times should be proportional to the pain evoked by performing the mental movement but not to current pain intensity. Patients' reaction times correlated with symptom duration and pain that would be evoked by executing a movement but not with pain intensity. Moseley proposed the existence of a “guarding-type” mechanism, affecting motor processes at the level of planning movements and the involvement of long-term changes in the cortical brain regions that participate in body representation. In the same line, Peltz et al. (2011) found that CRPS patients overestimated the size of their hand, and the degree correlated with disease duration, tactile discrimination, and neglect symptoms. Although not significant, we observed a tendency of a progressive increase in the mean passability ratio stratified by pain symptom duration. A larger sample size might evidence a significant relationship with symptom duration.

It is noteworthy that the pain distribution results show greater concentration of pain in the shoulders and cervical and lumbar regions. The question arises whether there is a relationship between the passability ratio and pain location. Since the body-scaled action-anticipation task specifically involves shoulder width, one could hypothesize that overestimation of body size is due to the concentration of pain in the shoulder area alone. We compared the mean passability ratio for subsamples of patients that had pain in different locations with the total fibromyalgia group (see supplementary material) and found a significantly higher mean ratio in subsamples with pain in the thighs, cervical, upper arms, shoulders, wrists, elbows, neck, and lumbar region. Given the small number of cases for some pain locations, it was not possible to perform a comparison. A further limitation was that patients felt pain in more than one location; therefore, we were unable to determine whether the fact of obtaining a higher passability ratio in a subsample presenting pain at a given location is exclusively related to the presence of pain at that location.

To assess the hypothesis that overestimation of body size was due to concentration of pain in the shoulder area alone, we compared the mean passability ratio of a subsample of patients who had no shoulder pain (NSP) with that of the total fibromyalgia group. The result indicated that the NSP subsample had a higher passability ratio than the fibromyalgia group, discarding that hypothesis (mean π_NSP_ ± SD: 1.72 ± 0.26; mean π_FM_ ± SD: 1.61 ± 0.26, *p* = 0.016). These results, together with the fact that body size overestimation correlates positively with the *Interference* dimension of the BPI but not with the *Severity* dimension or with current pain intensity, led us to consider that it was not current pain in a specific location that directly affected oversize estimation. Nevertheless, the relationship between body size overestimation and pain location warrants further investigation.

### Interoceptive sensitivity

Our second hypothesis was that fibromyalgia patients experience disrupted IS compared with controls. There was no difference in *IS*-values between groups for the heartbeat detection task, which does not support our hypothesis.

The correlation analysis over the whole sample showed no relationship between IS and the FIQ/SIQ, in agreement with the lack of difference in IS between groups. Likewise, there was no association between IS and the *Severity* and *Interference* dimensions of the BPI. Taken together, these results indicate that IS is not related to fibromyalgia symptoms.

In contrast to our findings, Duschek et al. (2015) found decreased IS in fibromyalgia patients and a negative linear association between IS and fibromyalgia symptom severity using a similar experimental paradigm. The difference may be due to an interaction between interoception and emotional variables. Dunn et al. (2010) argued that contradictory clinical evidence regarding interoception might be explained by an interaction with depression and anxiety. The authors applied the Clark and Watson (1991) tripartite model in which depression and anxiety are not considered monolithic typologies, but dimensional constructs that share a common component of negative affect differentiated by specific symptoms: anhedonia for depression and hyperarousal for anxiety. Assessing IS with the heartbeat detection task and symptoms with a short form of the Mood and Anxiety Symptom Questionnaire, Dunn et al. showed that the relationship between arousal and interoceptive accuracy weakened as anhedonia symptoms increased, suggesting interactions among interoception, depression, and anxiety (Dunn et al., 2010).

In the present work, fibromyalgia patients exhibited depression, anxiety, and stress as assessed by the DASS-21. Considering the IS score and mental health variables, an inverse association was observed: higher scores on depressive symptoms were coincident with lower IS, in agreement with previous studies (Pollatos et al., 2009; Terhaar et al., 2012). However, assessing groups individually, we found an inverse association between IS and depressive and stress symptoms in the fibromyalgia group, while the control group exhibited a positive correlation between the IS score and *Anxiety*. This contrast could suggest a different emotional-affective background in patients and controls, which could interact differently with IS. Taking into account the findings of Dunn et al. (2010) anhedonic and/or hyperarousal symptoms could have interacted with interoceptive performance, resulting in a lack of difference between the groups. A limitation of our study is that these symptoms were not specifically assessed.

No explanatory conclusions regarding IS can be extrapolated from the present findings. The interplay of emotional variables, particularly depressive and anxiety symptoms, between pain and IS in fibromyalgia, should be explored in future works using more complex models and larger participant samples.

### Interoceptive awareness

The MAIA total score did not differ between the fibromyalgia and control groups. Scores for *Noticing* were higher in the fibromyalgia group, suggesting patients are more aware of uncomfortable, comfortable, and neutral body sensations than controls. In addition, *Trusting* scores were lower among patients with fibromyalgia.

Notably, Cronbach's alpha estimates for *Not distracting* and *Not worrying* were low (α = 0.21; α = 0.39, respectively), indicating that these two dimensions cannot be reliably interpreted. Low Cronbach's alpha values were obtained for *Not Distracting* and *Not Worrying* in our evaluation of MAIA psychometric properties (α = 0.487; α = 0.402, respectively; Valenzuela-Moguillansky and Reyes-Reyes, 2015), suggesting cautious interpretation with respect to these dimensions and a need to verify the survey's Spanish translation.

An inverse relation was found between the total MAIA score and the FIQ/SIQ and the *Severity* and *Interference* dimensions of the BPI over the whole sample, indicating lower interoceptive awareness with a higher impact of fibromyalgia/any discomfort symptoms. In agreement with comparisons between groups, *Noticing* correlated positively with the FIQ/SIQ over the whole sample. On the other hand, *Self-regulation* and *Trusting* negatively associated with the FIQ/SIQ and both dimensions of the BPI, indicating that fibromyalgia/any discomfort symptoms are related to a reduced ability to regulate distress by attending to body sensations, as well as experiencing one's body as safe or trustworthy. These results suggest that while fibromyalgia patients exhibit greater awareness of uncomfortable, comfortable, and neutral body sensations, they cannot use this awareness to regulate distress. This idea is supported by correlations between the MAIA and mental health variables. The total MAIA score correlated negatively with *Depression* on the DASS-21, suggesting lower general interoceptive awareness with higher depressive symptoms. *Noticing* associated positively with the *Anxiety* and *Stress* dimensions of the DASS-21, indicating greater awareness of body sensations as anxiety or stress increase. *Self-regulation* associated negatively with the *Depression* and *Stress* dimensions of the DASS-21, indicating reduced ability to regulate distress by attending to body sensations as depression or stress increase. *Trusting* associated negatively with *Anxiety* and *Stress*, suggesting diminished experience of one's body as safe or trustworthy with elevated anxiety or stress.

The correlation between *Noticing* with *Anxiety* and *Stress* could be understood as expressing some form of “somatosensory amplification” (Barsky and Wyshak, 1990; Barsky et al., 1990; Cameron, 2002; Mailloux and Brener, 2002; De Berardis et al., 2007), described as a heightened attentional focus on the body, anxious vigilance of bodily signals, and self-focusing (as in hypochondriasis). This might explain the lower scores in *Trusting*. For fibromyalgia patients, bodily sensations are a source of anxiety and distress. Thus, it is consistent that body awareness is an alarm rather than an experience of non-judgmental acceptance of and connection with bodily sensations. Such body awareness can lead to a process of “objectification of body sensations,” in which body sensations are experienced as an object of perception rather than constituting the subject that perceives. Accordingly, bodily sensations are no longer part of the background of patients' embodied experience of the world; rather, they become a foreign object from which they need to protect themselves. Consequently, although attention to bodily sensations is increased, there is a concomitant process of taking distance and disconnection from body sensations, leaving the individual without bodily based emotional tools for self-regulation processes (Damasio, 2005). Such a process is coherent with fibromyalgia patients' reports of an “alienated” (Calsius et al., 2015) or “foreigner” body (Valenzuela-Moguillansky, 2013). This experience, different from the experience of “alienated” body in schizophrenia patients that directly expresses an “alienated” embodied self or disembodiment (Parnas and Handest, 2003; Fuchs and Schlimme, 2009; Parnas and Sass, 2010; Sestito et al., 2015a,b), expresses an aching body “as if” it was foreigner to the patient but experienced within a preserved sense of self. In this regard, the *embodied affectivity model* (Fuchs, 2013; Fuchs and Koch, 2014; Gaete and Fuchs, 2016) proposes that bodily resonance of emotions plays a key role in the experience of affects. Such model considers that without bodily resonance of emotions the experience of the world is devoid of meaning, as is the case of the bodily constriction observed in depressive patients, of which the so-called *Cotard's* syndrome is its main expression, or the case of alexithymia traits of somatoform or eating disorders. The negative association between the total MAIA score and *Depression* could indicate difficulties in patients' bodily resonance of emotions, as some authors have proposed (Brosschot and Aarsse, 2001; van Middendorp et al., 2008). In this regard, Hsu et al. (2010) treated fibromyalgia using Affective Self-Awareness (ASA), proposing that affects and how they are regulated (inhibition and avoidance or identification and expression) play a role in pain experience. They reported a significant reduction in pain severity, improved self-reported physical function, and a higher tender points threshold following ASA, applying mindfulness techniques toward breath, body, and emotions without judgment.

### Relationship between exteroceptive and interoceptive body awareness

Confirming our hypothesis, we observed a relationship between exteroceptive and interoceptive body awareness. The passability ratio and the IS score correlated negatively across the whole sample, meaning lower sensitivity to internal signals with higher passability ratios, i.e., higher the disruption of exteroceptive body awareness. To our knowledge, this is the first result showing that the estimation of body size relates to the perception of inner sensations. This result expands on Tsakiris et al. (2011) who report greater IS measured by the heartbeat detection task with reduced illusion of ownership of a rubber hand. In addition, through the MAIA we assessed an attitudinal disposition to being connected to internal states. There was a negative association between the passability ratio and *Body Listening* dimension of the MAIA, indicating a lower tendency to actively listen to the body for insight among subjects with higher passability ratios. These results suggest an interaction between mechanisms underlying the perception of our body in relation to space, sensibility to internal signals, and awareness of our inner state.

**Figure 2:**
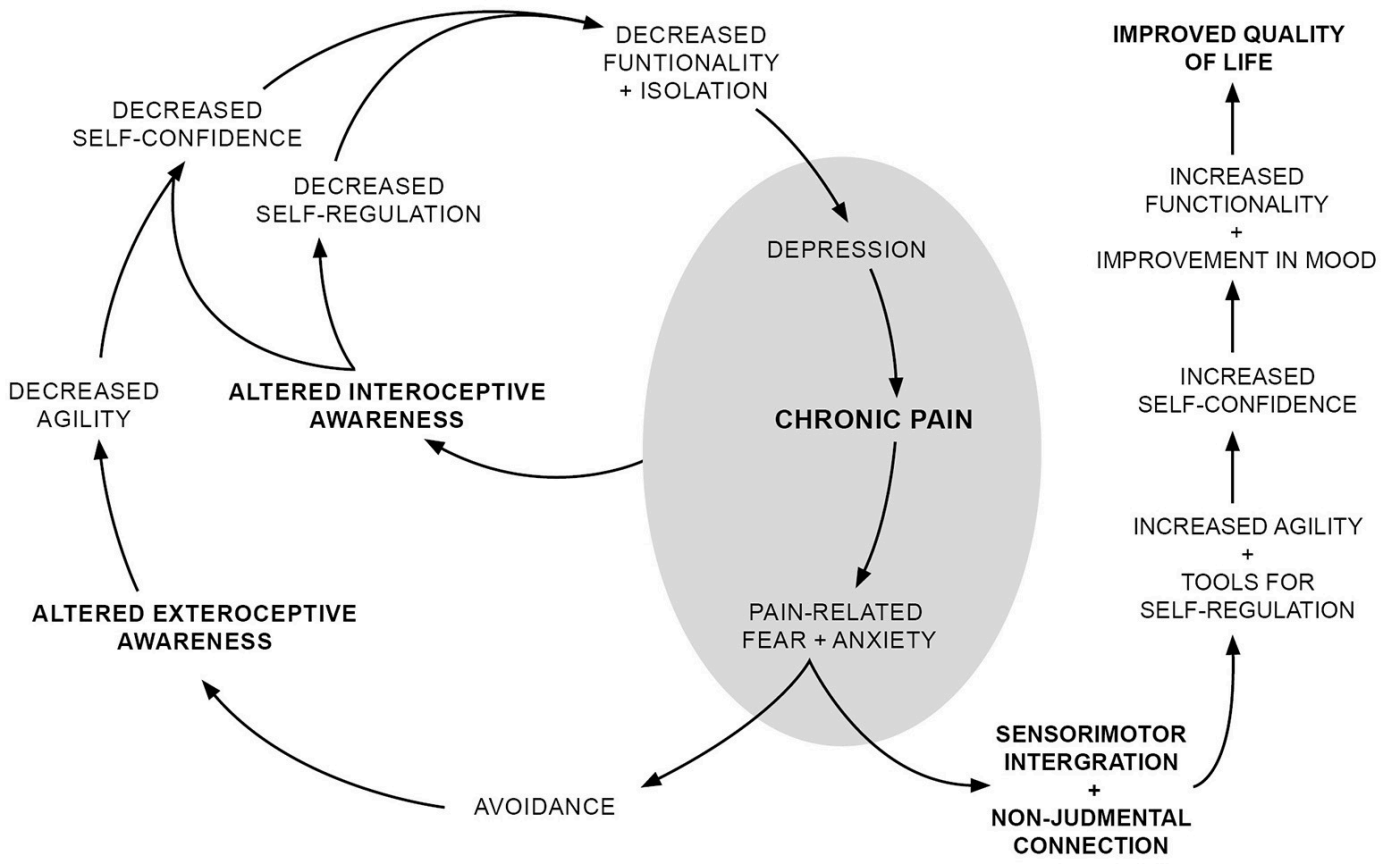
**Outline model of the relationship between pain, exteroceptive and interoceptive body awareness**.

Finally, we would like to relate our unique results in a schematic (Figure 2) inspired by the Vlaeyen and Linton fear-avoidance model (see Crombez et al., 2012; Vlaeyen and Linton, 2012, for an up to-date discussion), which was originally conceived to understand how acute injury pain becomes chronic. In such a context, rumination, and catastrophizing, cognitive aspects of pain-related fear, were considered as determinants in the evolution of the state of pain. In our work, we take as a point of departure a situation of chronic pain. Although catastrophizing and rumination are probably involved in aggravating pain in patients with fibromyalgia, we will not emphasize this aspect. We believe that considering the immediate behavioral aspect of pain-related fear—“it hurts, therefore I avoid it”—is sufficient to discuss possible interactions between exteroceptive and interoceptive body awareness and pain, which is the aim of our model.

We take as a starting point the situation of chronic pain that typically includes symptoms of depression and anxiety. As proposed by Vlaeyen and Linton (2000), pain-related fear promotes avoidance behaviors, which modifies patients' motor patterns. We proposed that the modification in motor patterns alter patients' exteroceptive body awareness, decreasing agility and physical dexterity, which is supported by the higher rate of falls and balance loss in people with fibromyalgia (Jones et al., 2009; Meireles et al., 2014). This experience of a clumsy body might lead to decreased confidence, as suggested by a negative correlation between the *Trusting* dimension of the MAIA and FIQ/SIQ scores. Lack of confidence in one's body might lead to decreased functionality and isolation; impacting social relationships and emotional wellbeing; and enhancing depression, anxiety, and pain. Such emotional states can impact interoceptive body awareness and foster objectification of body sensations. Here, attention to body sensations is coupled with a disconnection from them, contributing to decreased self-confidence and leaving the patient without bodily based emotional tools for self-regulation processes. These factors contribute to dysfunctionality and isolation, aggravating pain and patients' emotional state. In addition, the inverse relationship between the passability ratio and *Body listening* support the idea that exteroceptive and interoceptive body awareness are related; disconnection from bodily sensations might aggravate the distortion of exteroceptive body awareness and vice versa.

A two-pronged strategy aimed at re-establishing appropriate sensorimotor processing and enabling connection with emotions and bodily sensations in a non-judgmental manner is suggested to overcome these vicious cycles and improve patients' quality of life. A movement-based embodied contemplative practice such as yoga, the Feldenkrais method, or tai chi could be suitable to fulfill those objectives (Schmalzl and Kerr, 2016). Such practices can modify sensorimotor processing (Kerr et al., 2016) and foster non-judgmental connections with emotions and bodily sensations (Gard et al., 2014). This could help re-establish coherent exteroceptive body awareness and regaining familiarity with bodily sensations as part of patients' embodied subjectivity. In turn, coherent exteroceptive body awareness would improve patients' agility and self-confidence, and connection with bodily sensations would provide tools for emotional regulation, also improving self-confidence. Altogether, this would increase functionality, decreasing depression, and anxiety, and improving patient quality of life. The inverse relationship between the passability ratio and *Body listening* supports the idea that targeting both exteroceptive and interoceptive body awareness may be synergistic, enhancing the therapeutic effect of each dimension of the treatment.

Before concluding, we would like to refer to the relationship between interoceptive awareness and IS. Contrary to a dichotomized vision of interoceptive awareness and IS—one being adaptive and the other maladaptive—our results suggest these constructs share some aspects. Both the MAIA total score and IS score associated negatively with depression, indicating that these two aspects of interoception (a sense of self grounded in experiencing physical sensations in a non-judgmental way and accuracy in sensing an internal signal) decrease with higher depressive symptom burden. In addition, we found a positive association between the IS score and MAIA total score (Table 8). The following MAIA dimensions associated with the IS score were *Attention regulation, Emotional awareness*, and *Body listening*. Interestingly, these dimensions did not associate with pain or mental health variables, and there was no difference between groups. This is coherent with the lack of a difference in IS scores. Understanding the different modes of body awareness underlying the constructs of IS and interoceptive awareness, as well as the circumstances and individual characteristics in which these attentional modes might be adaptive or maladaptive, warrant further investigation.

**Table 8:** **Pearson correlation coefficient between the MAIA and IS score within the whole sample**.

**MAIA**	**IS score**
Noticing	0.183
Not distracting	0.079
Not worrying	0.095
Attention regulation	0.446^**^
Emotional awareness	0.416^**^
Self-regulation	0.209
Body listening	0.372^**^
Trusting	−0.022
MAIA total	0.382^**^

** *p < 0.001*.

In summary, the present findings more precisely define which aspects of body awareness are altered in fibromyalgia patients and how. We outlined a model highlighting the interaction between pain and exteroceptive and interoceptive aspects of body awareness. Movement-based embodied contemplative practices aimed at re-establishing sensorimotor integration and foster non-judgmental reconnection with bodily sensations are suggested to improve body confidence, functionality, and quality of life. Our results expand the scope of reflection regarding the relationship between body awareness and pain, including interoceptive and emotional aspects of the pain-body relationship.

## Author contributions

CVM conceived, designed and performed the study. ARR performed the statistical analysis and gave critical revision to the draft. MIG contributed with the interpretation of the data and gave critical revision to the draft.

## Funding

This work was supported by CONICYT (Comisión Nacional de Investigación Científica y Tecnológica-Chile)—PAI (National fellowship to support the return of researchers from abroad—project 82130040 to CVM.

### Conflict of interest statement

The authors declare that the research was conducted in the absence of any commercial or financial relationships that could be construed as a potential conflict of interest.

## Footnotes

^1^As explained in Method, the SIQ is equivalent to the FIQ but does not contain any reference to fibromyalgia; it was used for comparing the fibromyalgia and control groups.
