# Supplementary material for: Exteroceptive and Interoceptive Body-Self Awareness in Fibromyalgia Patients
Source: Front Hum Neurosci. 2017 Mar 13;11:117. doi: 10.3389/fnhum.2017.00117 (PMC5346579; doi:10.3389/fnhum.2017.00117)
Supplement: Supplementary file 1 [file Table1.DOCX]

Table 1. Change in mean passability ratio according to pain location and comparison with the means of the fibromyalgia and control groups.

| Localization of pain | *n* | Mean | *SD* | *p-value^a^* | |
| --- | --- | --- | --- | --- | --- |
|  |  |  |  | Fibromyalgia (*M*=1.61) | Control (*M*=1.46) |
| Hip | 5 | 2.03 | .35 |  | * |
| Thigh | 9 | 1.96 | .38 | * | ** |
| Head | 5 | 1.92 | .41 |  |  |
| Foot | 5 | 1.91 | .34 |  | * |
| Cervical | 16 | 1.90 | .28 | ** | ** |
| Upper arm | 10 | 1.90 | .38 | * | ** |
| Calf | 5 | 1.88 | .39 |  |  |
| Shoulders | 15 | 1.88 | .37 | * | ** |
| Wrist | 7 | 1.87 | .22 | * | ** |
| Upper Torax | 9 | 1.86 | .35 |  | ** |
| Elbow | 10 | 1.86 | .33 | * | ** |
| Neck | 7 | 1.86 | .23 | * | ** |
| Fore arm | 4 | 1.85 | .44 | -- | -- |
| Lumbar | 16 | 1.82 | .32 | * | ** |
| Ribs | 2 | 1.80 | .10 | -- | -- |
| Shin | 4 | 1.78 | .49 | -- | -- |
| Ankle | 10 | 1.78 | .28 |  | ** |
| Hand | 7 | 1.75 | .36 |  |  |
| Sacrum | 4 | 1.74 | .18 | -- | -- |
| Knee | 12 | 1.73 | .28 |  | ** |
| Buttocks | 9 | 1.72 | .15 |  | ** |
| Chest | 4 | 1.68 | .28 | -- | -- |
| Lower Torax | 7 | 1.66 | .21 |  | * |
| Jaw | 2 | 1.61 | .17 | -- | -- |
| Face | 1 | 1.49 | - | -- | -- |
| Belly | 1 | 1.49 | -_ | -- | -- |

*a, Non-parametric test; *p<0.05; **p<0.01; --* *Not calculated given n <5*
